# Synergistic impact of Composite Dietary Antioxidant Index and physical activity on fatty liver disease

**DOI:** 10.3389/fnut.2024.1486700

**Published:** 2024-11-05

**Authors:** Linxiao Gao, Haoyu Fang, Zhibo Zhao, Wen Luo, Jianping Gong, Junhua Gong

**Affiliations:** Department of Hepatobiliary Surgery, The Second Affiliated Hospital of Chongqing Medical University, Chongqing, China

**Keywords:** Composite Dietary Antioxidant Index, nonalcoholic fatty liver disease, metabolic-associated fatty liver disease, NHANES, inflammatory biomarkers, physical activity

## Abstract

**Background:**

The relationship between dietary antioxidants and fatty liver disease remains a topic of debate. This study aimed to examine the association between the Composite Dietary Antioxidant Index (CDAI) and nonalcoholic fatty liver disease (NAFLD)/metabolic-associated fatty liver disease (MAFLD).

**Methods:**

The study analyzed data from the 2003–2018 cycles of the National Health and Nutrition Examination Survey. The study included 16,321 individuals aged 20–85 years. Food and nutrient intake data were based on the 24-h recall method. Multivariate logistic regression models were employed to examine the relationship between CDAI and NAFLD/MAFLD.

**Results:**

In the fully adjusted multivariate logistic regression model, CDAI demonstrated a significant negative correlation with NAFLD and MAFLD. Mediation analysis showed that inflammatory factors partially mediated the relationship between CDAI and NAFLD/MAFLD prevalence. The combination of high CDAI levels with effective physical activity was associated with a greater reduction in NAFLD/MAFLD prevalence than high CDAI levels alone.

**Conclusion:**

Our study highlighted a negative association between CDAI and NAFLD/MAFLD, mediated by inflammatory factors. Additionally, participants with characteristics of active physical activity and high levels of CDAI were more strongly correlated with the reduced prevalence of NAFLD/MAFLD. Further research in clinical cohorts should be conducted to comprehensively investigate the impact of CDAI on NAFLD/MAFLD prevalence.

## 1 Introduction

Nonalcoholic fatty liver disease (NAFLD) is the most common liver disease worldwide, with a global prevalence within the limits of 25–35% (1, 2), and is the first cause of chronic liver disease (3). The progression of NAFLD culminates in nonalcoholic steatohepatitis, which, in turn, can progress to fibrosis, cirrhosis, and ultimately hepatocellular carcinoma (4). In early 2020, several researchers proposed changing the term NAFLD to metabolic-associated fatty liver disease (MAFLD) to reflect a strong pathophysiological connection between steatosis and metabolic dysfunction (5). Despite the rising tide of the global burden of NAFLD/MAFLD, effective pharmacological agents are limited (6). Resmetirom is a selective thyroid hormone receptor-beta agonist that is orally active and targeted toward the liver, which is specifically developed to improve hepatic fat metabolism and reduce lipotoxicity (7). Of the more than 400 ongoing clinical trials, only resmetirom has been approved by FDA (8, 9).

NAFLD results from a multifaceted interplay of molecular factors, including lipid metabolism, inflammation, oxidative stress, and mitochondrial dysfunction (10). The current disease management plan depends mainly on lifestyle interventions (11). The holistic approach involving a balanced lifestyle encompassing diet, physical activity (PA), sleep, and other factors is recommended to prevent NAFLD/MAFLD (12, 13). Several different diet patterns, including the Mediterranean diet, ketogenic diet, low-calorie and low-fat diet have been considered as potential strategies for preventing and treating NAFLD/MAFLD (14–16). In these diet patterns, some antioxidants such as monounsaturated oils, fibers, and phytosterols have been found to help reduce liver steatosis (17). Relevant studies have confirmed that antioxidants such as vitamin E, and vitamin C in the diet are related to a decreased risk of NAFLD/MAFLD (18, 19).

Antioxidants play a crucial role in neutralizing the harmful impacts of reactive oxygen species (ROS) generated during normal cellular activities (20). Oxidative stress centrally contributes to hepatic steatosis, insufficient antioxidant intake may destroy the redox balance (21, 22). A case–control study in Iran indicated that a higher dietary total antioxidant capacity (DTAC) was associated with a lower risk of NAFLD (23). Similarly, Vahid et al. have found the dietary antioxidant index (DAI) calculated based on vitamins A, C, E, selenium, manganese, and zinc, is negatively related to the incidence of NAFLD (24). However, DTAC can be influenced by various factors such as food preparation and cooking methods, and DAI ignored some non-nutritive antioxidants such as carotenoids.

The Composite Dietary Antioxidant Index (CDAI) is a scoring system that represents the comprehensive profile of dietary antioxidant intake (including vitamins A, C, E, selenium, zinc, and carotenoids) developed by Wright et al. (25). It has been proposed to be associated with many human diseases, such as depression, hypertension, and osteoporosis (26–28). Two cross-sectional studies included similar participants from 2 cycles of the National Health and Nutrition Examination Survey (NHANES) database, have found CDAI is negatively associated with metabolic dysfunction-associated steatotic liver disease (MASLD) (29, 30). However, the dose–response relationship was not exactly consistent, moreover, the association between CDAI and NAFLD/MAFLD was not explored. Therefore, we carried out an observational study analyzing the NHANES database to delve into the potential association between CDAI and NAFLD/MAFLD.

## 2 Materials and methods

### 2.1 Study population

The NHANES is a cross-sectional survey evaluating the health and nutritional status of both adults and children in the US initiated by the Centers for Disease Control and Prevention. It has been updated every 2 years since 1999 and was approved by the National Center for Health Statistics Research Ethics Review Board. All participants provided informed consent (31). This study used eight NHANES 2-year cycles (2003–2018). This study follows the Strengthening the Reporting of Observational Studies in Epidemiology reporting guideline.

The exclusion criteria were as follows: (1) missing information about dietary data on the first day; (2) liver disease due to other causes (liver cancer, autoimmune hepatitis, hepatitis B, hepatitis C); (3) missing information about other variables such as age, sex, race, body mass index (BMI), education level, and smoking status; (4) participants with extreme energy intake (less than 800 kcal or more than 4,000 kcal for male, less than 500 kcal or more than 3,500 kcal for female); (5) participants under 20 years of age. Supplementary Figure 1 shows the details of the inclusion and exclusion processes used in this study.

### 2.2 Calculation of CDAI

The food and nutrient intake data of the NHANES participants were acquired through a 24-h dietary recall interview. The initial dietary recall occurred in person at a mobile inspection center, and the second was conducted 3–10 days later via telephone. The Food and Nutrient Database for Dietary Studies of the United States Department of Agriculture was used to calculate the nutritional components in foods (32). The CDAI composed of six vitamins and minerals (vitamins A, C, E, carotenoids, selenium, and zinc), was calculated as follows:

$$\mathrm{CDAI}=\overset{n=6}{\underset{i=1}{\sum }}\frac{x_{i}-\mu_{i}}{S_{i}}$$

In this formula, x_i_ is the daily intake of antioxidants, μ_i_ is the mean of x_i_, and S_i_ is the standard deviation of μ_i_.

### 2.3 Assessment of NAFLD and MAFLD

NAFLD is a pathological syndrome characterized by abnormal fat accumulation in the liver, defined as any degree of steatosis excluding patients resulting from excessive alcohol consumption or other liver diseases such as viral hepatitis, autoimmune hepatitis, and liver cancer (33). Excessive alcohol consumption was defined as an average of >20 g/day for males and >10 g/day for females (34). MAFLD was defined based on hepatic steatosis and any of the three criteria listed below: overweight/obese (BMI ≥ 25 kg/m^2^), type 2 diabetes, or metabolic dysregulation. Metabolic dysregulation is defined as the presence of at least two of the following: (1) waist circumference ≥102 cm for male and ≥88 cm for female; (2) blood pressure ≥130/85 mmHg or on anti-hypertensive therapy; (3) plasma triglycerides ≥1.70 mmol/L or on lipid-lowering therapy; (4) serum high-density lipoprotein cholesterol <1.0 mmol/L for male and <1.3 mmol/L for female or specific drug treatment; (5) diabetes or pre-diabetes (fasting glucose level 5.6–6.9 mmol/L, or 2-h post-load glucose level 7.8–11.0 mmol or HbA1c 5.7–6.4%); (6) serum hypersensitivity C-reactive protein level >2 mg/L; (7) homeostasis model assessment insulin resistance score ≥2.5 (35).

Serological-based noninvasive tests, such as the Fatty Liver Index (FLI) and United States Fatty Liver Index (USFLI), are widely embraced in clinical settings as surrogate markers for stratifying the risks associated with hepatic steatosis (9, 36). The FLI and USFLI were calculated as follows:

$$FLI=\frac{e^{x}}{1+e^{x}}\times 100$$

$$\mathrm{USFLI}=\frac{e^{y}}{1+e^{y}}\times 100$$

$$\begin{array}{l} x=0.953\times \ln TG+0.139\times BMI+0.718\\ \times \ln GGT+0.053\times \mathrm{waist circumfernce}-15.745 \end{array}$$

$$\begin{array}{l} y=0.3458\times \mathrm{Mexican American}-0.8073\times \\ \mathrm{Non-Hispanic Black}+0.0093\times \mathrm{age}+0.6151\\ \times \ln GGT+0.0249\times \mathrm{waist circumfernce}\\ +1.1792*\ln\mathrm{insulin}+0.8242\times \ln\mathrm{glucose}-14.7812 \end{array}$$

In this formula, “Non-Hispanic Black” and “Mexican American” have a value of 1 if the participant is of that ethnicity and 0 if not of that ethnicity (37). Subjects were defined as having hepatic steatosis if their FLI score ≥ 60 or their USFLI score ≥ 30 (38).

### 2.4 Measurement of inflammatory biomarkers

In this study, the inflammatory biomarkers of the subjects participating in the examination were white blood cell (WBC) count, neutrophils count, lymphocytes count, and C-reactive protein. According to the NHANES 2003–2018 cycle protocol, the Beckman Coulter method of counting and sizing, in combination with an automatic diluting and mixing device for sample processing, was applied to count blood cells from the peripheral blood samples obtained from the NHANES Mobile Examination Center. For the processing of C-reactive protein, latex-enhanced nephelometry with particle-enhanced assays was used for quantitation.

### 2.5 Covariates

The demographic variables included age (years); sex (male/female); race (Mexican American, Other Hispanic, Non-Hispanic White, Non-Hispanic Black, or other races, including Multiracial); education level (<high school, ≥high school); and poverty income ratio (low level, middle level, high level). The questionnaire data included recreational activity level (vigorous activities, moderate activities, and no activities. Vigorous activities were defined as activities that cause large increases in breathing or heart rate like running or basketball for at least 10 min continuously. Moderate activities were defined as activities that cause a small increase in breathing or heart rate such as brisk walking, bicycling, swimming, or volleyball for at least 10 min continuously), and active PA was defined as vigorous or moderate activities. Smoking status (never smoker, former smoker, current smoker).

### 2.6 Statistical analysis

To minimize the potential biases, after excluding data with missing covariates, baseline demographic characteristics between included and excluded groups were compared using standardized differences (differences <10% were regarded as negligible) (39) (Supplementary Table 1). The baseline characteristics of all included participants were described by median values and interquartile range (IQR) (continuous variables; expressed as the median [IQR]) or proportions (categorical variables; expressed as *N* [%]). Baseline characteristics were compared using an independent sample t-test for continuous variables and the χ^2^ test for categorical variables. Considering the intricate probability cluster design of the NHANES, all the statistical analyses in this study incorporated weights. Weighted multivariable-adjusted logistic regression was employed to investigate the association between CDAI and NAFLD/MAFLD, with results presented as odds ratios (OR) and 95% confidence intervals (CI). As recommended, covariates were selected for multivariable analyses based on our causal understanding of the existing literature rather than the statistical criteria (40). Model 1 lacked confounder adjustments; Model 2 was adjusted for demographic covariates (age, gender and race); Model 3 was further adjusted for family income, education level, smoking status, activity level, and energy intake, as these covariates have been regarded as the risk factors for hepatic steatosis (41–43). The results were validated by these different models to ensure the robustness of the findings.

Multivariate-adjusted restricted cubic spline (RCS) analysis and two-piecewise linear regression for threshold effect analysis were utilized to explore the nonlinear relationship. RCS is a flexible nonlinear model that accurately represents interactions and varying rates of change, then threshold effect analysis was conducted to identify potential cut-off points or thresholds. It is noted that the number of knots was selected based on the Akaike’s information criterion (AIC), with the lowest AIC values indicating the best-fitted model. Likelihood ratio test was used to test the overall significance of the two-piecewise linear regression model. Mediation analysis was conducted to assess whether inflammatory factors mediated the association between CDAI and NAFLD/MAFLD prevalence, providing statistical support for mechanistic analysis. Stratified analyses were conducted to assess potential moderating effects of age, sex, race, family income, education level, smoking status, and activity level. Weighted multivariable-adjusted logistic regression assessed joint associations of CDAI and PA with NAFLD/MAFLD. Then, the population attributable fraction (PAF) was used to estimate the proportion of NAFLD/MAFLD which could be avoided if exposure (CDAI and/or PA) were eliminated. The PAF was widely used to measure the disease burden attributable to a given risk factor, and was calculated by the “AF” package in R software (44). All the data analyses were performed with R software (version 4.2.0). *p* < 0.05 indicated statistical significance.

## 3 Results

### 3.1 General characteristics of NHANES

Among 16,321 eligible adults with complete data on CDAI and NAFLD/MAFLD, the mean (IQR) age was 45 (32, 45) years, and 8,118 (50%) were female; 2,384 (7.2%) of participants were Mexican American, 3,143 (9.5%) of participants were Non-Hispanic Black, 8,025 (73%) of participants were Non-Hispanic White, and 2,769 (11%) of participants identified as other race or ethnicity; 5,591 (34%) were defined as overweight and 5,788 (34%) were defined as obesity. Among the included participants, 4,353 (27%) were defined as NAFLD, and 5,092 (31%) were defined as MAFLD. The baseline characteristics compared by NAFLD/MAFLD are shown in Table 1. Participants with NAFLD/MAFLD were more likely to be older, male, had lower income, less educated, more likely to smoke, engaged in less activity, and had lower CDAI levels. The characteristics of study population according to quartiles of CDAI were shown in Supplementary Table 2.

**Table 1:** Characteristics of the included participants from NHANES 2003–2018.

Characteristic	Overall	NAFLD			MAFLD		
	*N* = 16,321 ^1^	Non-NAFLD, *N* = 11,968 ^1^	NAFLD, *N* = 4,353 ^1^	*p* Value^2^	Non-MAFLD, *N* = 11,229 ^1^	MAFLD, *N* = 5,092 ^1^	*p* Value^2^
Age (years)	45 (32, 58)	42 (30, 55)	52 (39, 64)	**<0.001**	42 (30, 55)	52 (38, 63)	**<0.001**
Sex (%)				**<0.001**			**<0.001**
Female	8,118 (50%)	6,195 (52%)	1,923 (43%)		5,990 (54%)	2,128 (41%)	
Male	8,203 (50%)	5,773 (48%)	2,430 (57%)		5,239 (46%)	2,964 (59%)	
Poverty income ratio	3.25 (1.64, 5.00)	3.41 (1.74, 5.00)	2.76 (1.41, 4.86)	**<0.001**	3.40 (1.74, 5.00)	2.92 (1.44, 5.00)	**<0.001**
Education level (%)				**<0.001**			**<0.001**
<High school	3,367 (13%)	2,130 (12%)	1,237 (18%)		1,984 (11%)	1,383 (18%)	
≥High school	12,954 (87%)	9,838 (88%)	3,116 (82%)		9,245 (89%)	3,709 (82%)	
Race/ethnicity (%)				**<0.001**			**<0.001**
Mexican American	2,384 (7.2%)	1,352 (5.6%)	1,032 (12%)		1,197 (5.4%)	1,187 (12%)	
Non-Hispanic Black	3,143 (9.5%)	2,634 (11%)	509 (6.1%)		2,546 (11%)	597 (6.0%)	
Non-Hispanic White	8,025 (73%)	5,938 (73%)	2,087 (71%)		5,541 (73%)	2,484 (72%)	
Other/Multiracial	2,769 (11%)	2,044 (11%)	725 (11%)		1,945 (11%)	824 (10%)	
BMI (kg/m^2^)				**<0.001**			**<0.001**
<25.0	4,942 (33%)	4,763 (42%)	179 (3.5%)		4,743 (45%)	199 (3.5%)	
Overweight	5,591 (34%)	4,426 (37%)	1,165 (25%)		4,177 (37%)	1,414 (26%)	
Obesity	5,788 (34%)	2,779 (21%)	3,009 (72%)		2,309 (18%)	3,479 (71%)	
Activity level (%)				**<0.001**			**<0.001**
No activities	5,904 (30%)	3,732 (26%)	2,172 (45%)		3,425 (25%)	2,479 (43%)	
Moderate activities	6,134 (40%)	4,621 (41%)	1,513 (38%)		4,342 (41%)	1,792 (39%)	
Vigorous activities	4,283 (30%)	3,615 (34%)	668 (17%)		3,462 (34%)	821 (18%)	
Smoking status (%)				**<0.001**			**<0.001**
Current smoker	3,338 (21%)	2,593 (22%)	745 (16%)		2,385 (22%)	953 (18%)	
Former smoker	4,140 (26%)	2,787 (24%)	1,353 (32%)		2,510 (23%)	1,630 (33%)	
Never smoker	8,843 (53%)	6,588 (54%)	2,255 (52%)		6,334 (55%)	2,509 (49%)	
Energy (kcal)	2,031 (1,576, 2,652)	2,042 (1,582, 2,667)	1,977 (1,525, 2,584)	**0.008**	2,016 (1,574, 2,645)	2,062 (1,584, 2,660)	**0.4**
WBC (1,000 cells/uL)	6.40 (5.40, 7.70)	6.20 (5.20, 7.40)	7.10 (6.00, 8.40)	**<0.001**	6.10 (5.20, 7.40)	7.10 (6.00, 8.40)	**<0.001**
Hb (g/dL)	14.50 (13.50, 15.50)	14.40 (13.50, 15.40)	14.80 (13.70, 15.70)	**<0.001**	14.30 (13.40, 15.40)	14.80 (13.80, 15.80)	**<0.001**
PLT (1,000 cells/uL)	242 (206, 282)	241 (207, 280)	246 (204, 290)	0.3	241 (207, 280)	246 (204, 288)	0.5
GGT (IU/L)	19 (13, 28)	17 (12, 24)	27 (20, 43)	**<0.001**	16 (12, 22)	28 (20, 45)	**<0.001**
TB (umol/L)	12.0 (8.6, 15.4)	12.0 (8.6, 15.4)	12.0 (8.6, 13.7)	**<0.001**	12.0 (8.6, 15.4)	12.0 (8.6, 13.7)	**<0.001**
ALP (IU/L)	63 (52, 78)	61 (51, 75)	70 (58, 86)	**<0.001**	61 (50, 74)	69 (57, 84)	**<0.001**
Hs-CRP (mg/L)	1.8 (0.8, 4.2)	1.4 (0.6, 3.1)	3.1 (1.6, 6.8)	**<0.001**	1.3 (0.6, 3.0)	3.1 (1.4, 6.5)	**<0.001**
Vitamin C (mg)	64 (31, 117)	67 (33, 120)	55 (27, 107)	**<0.001**	67 (33, 121)	55 (27, 107)	**<0.001**
Vitamin A (ug)	550 (340, 825)	557 (342, 835)	531 (332, 803)	0.10	557 (341, 834)	534 (336, 809)	0.2
Vitamin E (mg)	7.4 (5.0, 10.4)	7.5 (5.1, 10.7)	7.0 (4.7, 9.7)	**<0.001**	7.5 (5.1, 10.7)	7.1 (4.8, 9.8)	**<0.001**
Carotenoids (mcg)	1,354 (525, 3,460)	1,423 (542, 3,648)	1,146 (480, 2,996)	**<0.001**	1,418 (535, 3,644)	1,211 (501, 3,089)	**<0.001**
Zinc (mg)	10.8 (7.8, 14.8)	10.9 (7.9, 14.8)	10.7 (7.6, 14.7)	0.3	10.8 (7.8, 14.7)	10.9 (7.8, 14.9)	0.5
Selenium (mcg)	108 (80, 143)	108 (80, 143)	109 (80, 144)	0.8	107 (79, 142)	112 (82, 146)	**0.024**
CDAI	−0.3 (−2.3, 2.2)	−0.2 (−2.3, 2.3)	−0.6 (−2.4, 1.6)	**0.001**	−0.2 (−2.3, 2.3)	−0.5 (−2.4, 1.8)	**0.049**

1 Median (IQR) for continuous; *n* (%) for categorical.

2 Wilcoxon rank-sum test for complex survey samples; chi-squared test with Rao & Scott’s second-order correction.

NAFLD, nonalcoholic fatty liver disease; MAFLD, metabolic-associated fatty liver disease; BMI, body mass index; WBC, white blood cell; Hb, hemoglobin; PLT, platelet; GGT, gamma glutamyl transferase; TB, total bilirubin; ALP, alkaline phosphatase; Hs-CRP, high sensitive C-reactive protein; CDAI, composite dietary antioxidant index. The bold values signify statistical significance at *p* < 0.05.

### 3.2 Association of CDAI and its components with the risk of NAFLD/MAFLD

The weighted multivariable logistic regression models were performed to investigate the association between CDAI with NAFLD/MAFLD. As shown in Table 2, after adjusting for multiple variables in model 3, the OR for NAFLD was 0.95 (95% CI, 0.91–1.00, *p* = 0.040) in the fourth quartile of CDAI compared to the reference group (*p* for trend = 0.030). Similarly, compared to the reference group, the fourth quartile of CDAI (OR, 0.93; 95% CI, 0.89–0.97, *p* = 0.002) was significantly related to the decreased prevalence of MAFLD. These results were robust across all models.

**Table 2:** The relationship between CDAI and NAFLD/MAFLD.

		Model 1^2^ OR (95% CI)	*p* Value	Model 2^3^ OR (95% CI)	*p* Value	Model 3^4^ OR (95% CI)	*p* Value
NAFLD	CDAI (continuous)	0.97 (0.95, 0.99)	**<0.001**	0.95 (0.93, 0.97)	**<0.001**	0.97 (0.94, 0.99)	**0.017**
	Quartile of CDAI						
	Q1	Ref.^1^		Ref.		Ref.	
	Q2	1.00 (0.97, 1.03)	0.994	0.98 (0.95, 1.01)	0.246	1.00 (0.96, 1.03)	0.785
	Q3	0.97 (0.94, 1.01)	0.157	0.96 (0.93, 0.99)	**0.012**	0.98 (0.95, 1.02)	0.257
	Q4	0.95 (0.91, 0.98)	**0.006**	0.92 (0.89, 0.95)	**<0.001**	0.95 (0.91, 1.00)	**0.040**
	*p* for trend	**0.003**		**<0.001**		**0.030**	
MAFLD	CDAI (continuous)	0.98 (0.96, 1.00)	**0.028**	0.96 (0.94, 0.98)	**<0.001**	0.95 (0.93, 0.98)	**<0.001**
	Quartile of CDAI						
	Q1	Ref.		Ref.		Ref.	
	Q2	1.02 (0.98, 1.06)	0.376	0.99 (0.96, 1.03)	0.754	1.00 (0.96, 1.04)	0.917
	Q3	0.99 (0.95, 1.04)	0.776	0.97 (0.93, 1.01)	0.116	0.97 (0.93, 1.02)	0.219
	Q4	0.97 (0.93, 1.01)	0.112	0.93 (0.89, 0.96)	**<0.001**	0.93 (0.89, 0.97)	**0.002**
	*p* for trend	0.074		**<0.001**		**0.001**	

1 Ref, reference.

2 Model 1 was non-adjusted model.

3 Model 2 was adjusted for age, gender, and race.

4 Model 3 was adjusted for age, gender, race, activity level, educational level, family income, energy intake, and smoking.

NAFLD, nonalcoholic fatty liver disease; MAFLD, metabolic-associated fatty liver disease; CDAI, composite dietary antioxidant index; OR, odds ratio; CI, confidence interval. The bold values signify statistical significance at *p* < 0.05.

Additional analyses were carried out to explore the association between six components of CDAI and NAFLD/MAFLD. Supplementary Table 3 reveals a negative relationship between log vitamin E (OR, 0.55; 95% CI, 0.38–0.79, *p* = 0.001) and log carotenoids (OR, 0.76; 95% CI, 0.65–0.88, *p* < 0.001) with NAFLD. Similarly, log vitamin C (OR, 0.81; 95% CI, 0.69–0.95, *p* = 0.012) and log vitamin E (OR, 0.46; 95% CI, 0.33–0.64, *p* < 0.001) were negatively associated with MAFLD (Supplementary Table 4). Multivariate adjusted RCS demonstrated a decreased risk of NAFLD/MAFLD with the intake of log vitamin C, log vitamin E, log carotenoids, and log zinc (Supplementary Figures 2, 3).

### 3.3 RCS curve plotting and threshold effect analysis

**Figure 1:**
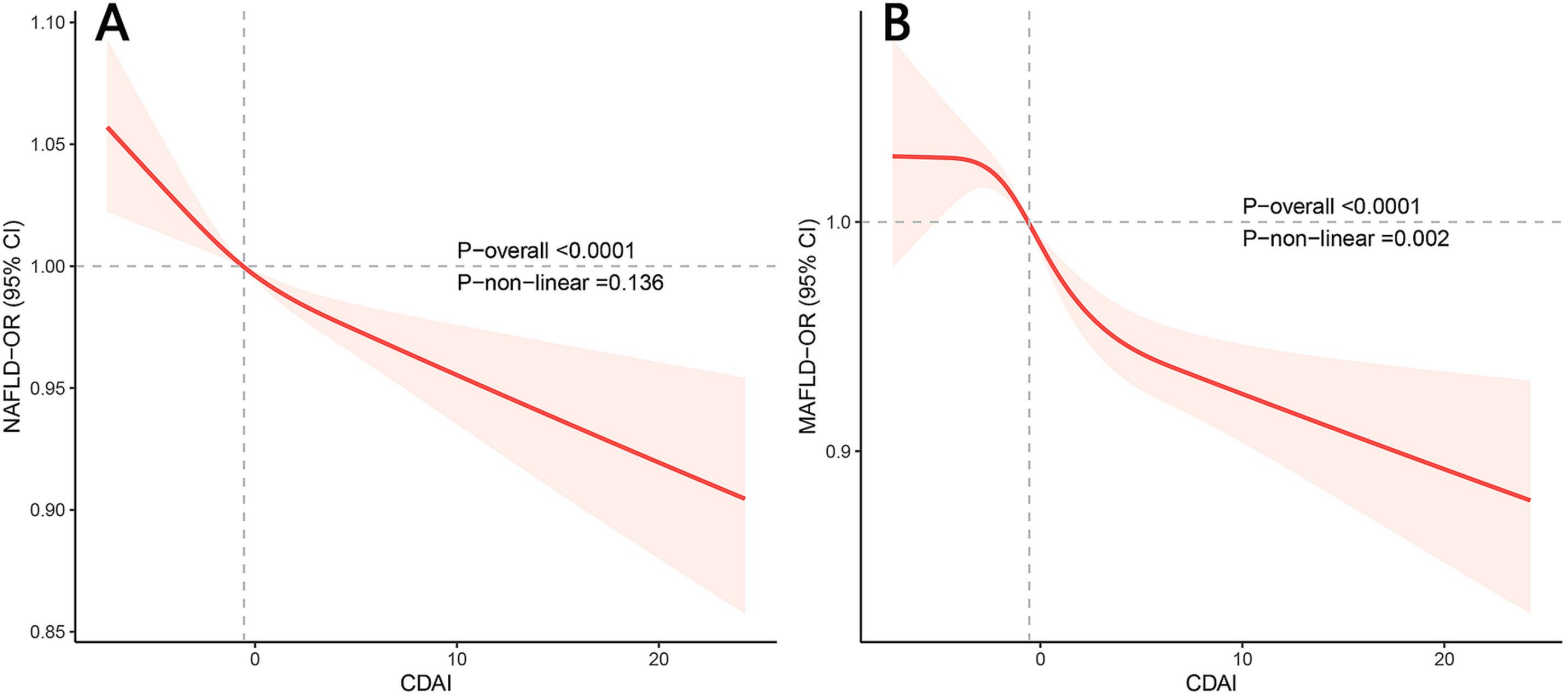
Restricted cubic spline plots of the association between CDAI and NAFLD/MAFLD prevalence. (A) NAFLD; (B) MAFLD. Adjusted for age, gender, race, activity level, educational level, family income, energy intake, and smoking. OR, odds ratio; CI, confidence interval; NAFLD, nonalcoholic fatty liver disease; MAFLD, metabolic-associated fatty liver disease; CDAI, composite dietary antioxidant index.

Figure 1 exhibits the dose–response relationships between CDAI and NAFLD/MAFLD. After multivariable adjustment, significant nonlinear associations were observed between CDAI and MAFLD (*p* for nonlinear = 0.002) using restricted cubic splines (*p* for overall <0.001). However, there was no significant nonlinear relationship between CDAI and NAFLD (*p* for nonlinear = 0.136; *p* for overall <0.001). A threshold effect analysis of CDAI on NAFLD/MAFLD was further performed by the two-piecewise linear regression. As shown in Supplementary Table 5, for MAFLD, the inflection point of CDAI was 2.792. Each unit increase of CDAI correlated with a 3% decrease of the risk of MAFLD below 2.792, with no statistically significant relationship above this threshold. However, the threshold effect for NAFLD was not significant (P for log-likelihood ratio > 0.05).

### 3.4 Mediation by inflammatory biomarkers

**Figure 2:**
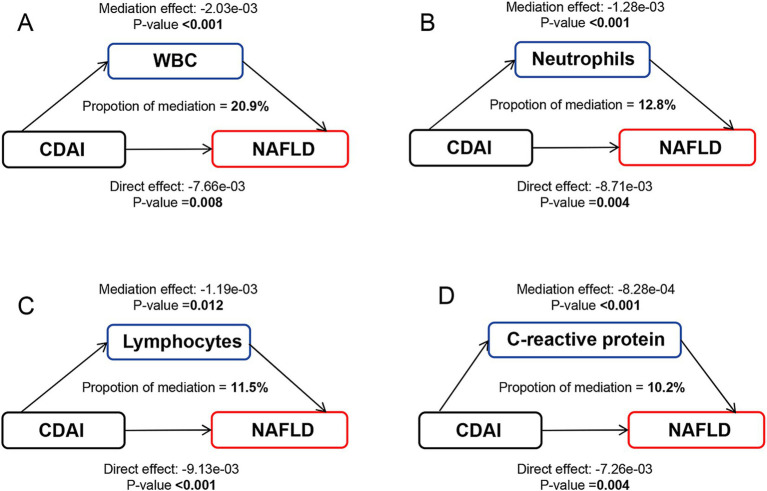
Path diagram of the mediation analysis of inflammatory biomarkers on the relationship between CDAI and NAFLD. The graphs in panel (A–D) represented the mediating role of WBC, neutrophils, lymphocytes, and C-reactive protein, respectively. NAFLD, nonalcoholic fatty liver disease; CDAI, composite dietary antioxidant index; WBC, white blood cell.

**Figure 3:**
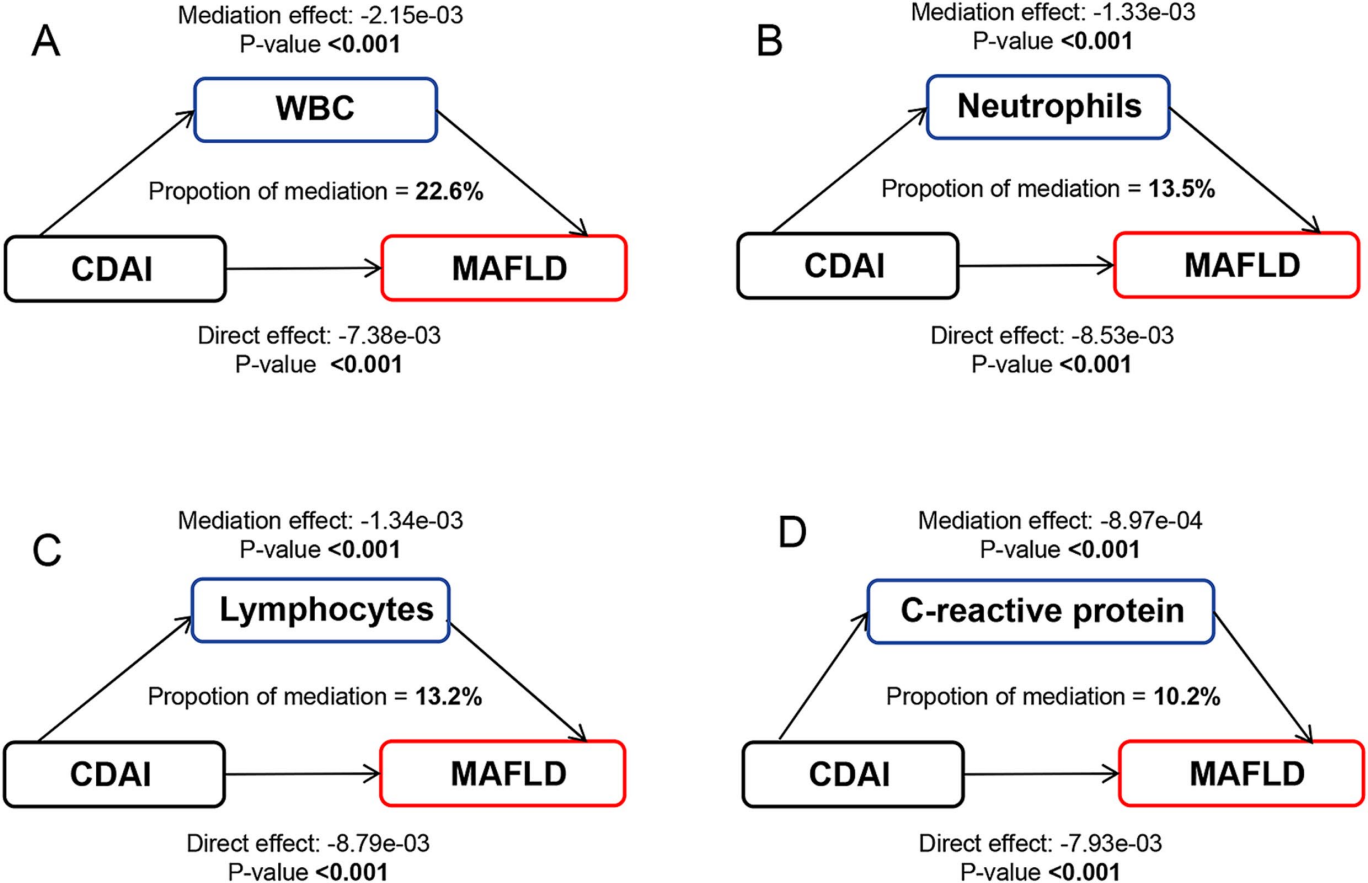
Path diagram of the mediation analysis of inflammatory biomarkers on the relationship between CDAI and MAFLD. The graphs in panel (A–D) represented the mediating role of WBC, neutrophils, lymphocytes, and C-reactive protein, respectively. MAFLD, metabolic-associated fatty liver disease; CDAI, composite dietary antioxidant index; WBC, white blood cell.

A significant negative relationship between CDAI and inflammatory biomarkers was found by the weighted multiple linear regression model (Supplementary Table 6). Mediation analyses were conducted to explore the mediating effect of inflammatory biomarkers. As shown in Figure 2, all four inflammatory biomarkers significantly mediated the association between CDAI and NAFLD. Specifically, CDAI reduced the prevalence of NAFLD by lowering the levels of these inflammatory factors, with WBC, neutrophils, lymphocytes, and C-reactive protein accounted for 20.9, 12.8, 11.5, and 10.2% of the association, respectively (all *p* for mediation <0.05). A similar pattern was observed for MAFLD, with WBC, neutrophils, lymphocytes, and C-reactive protein explaining 22.6, 13.5, 13.2, and 10.2% of the association, respectively (all *p* for mediation <0.05) (Figure 3).

### 3.5 Stratified analysis

**Figure 4:**
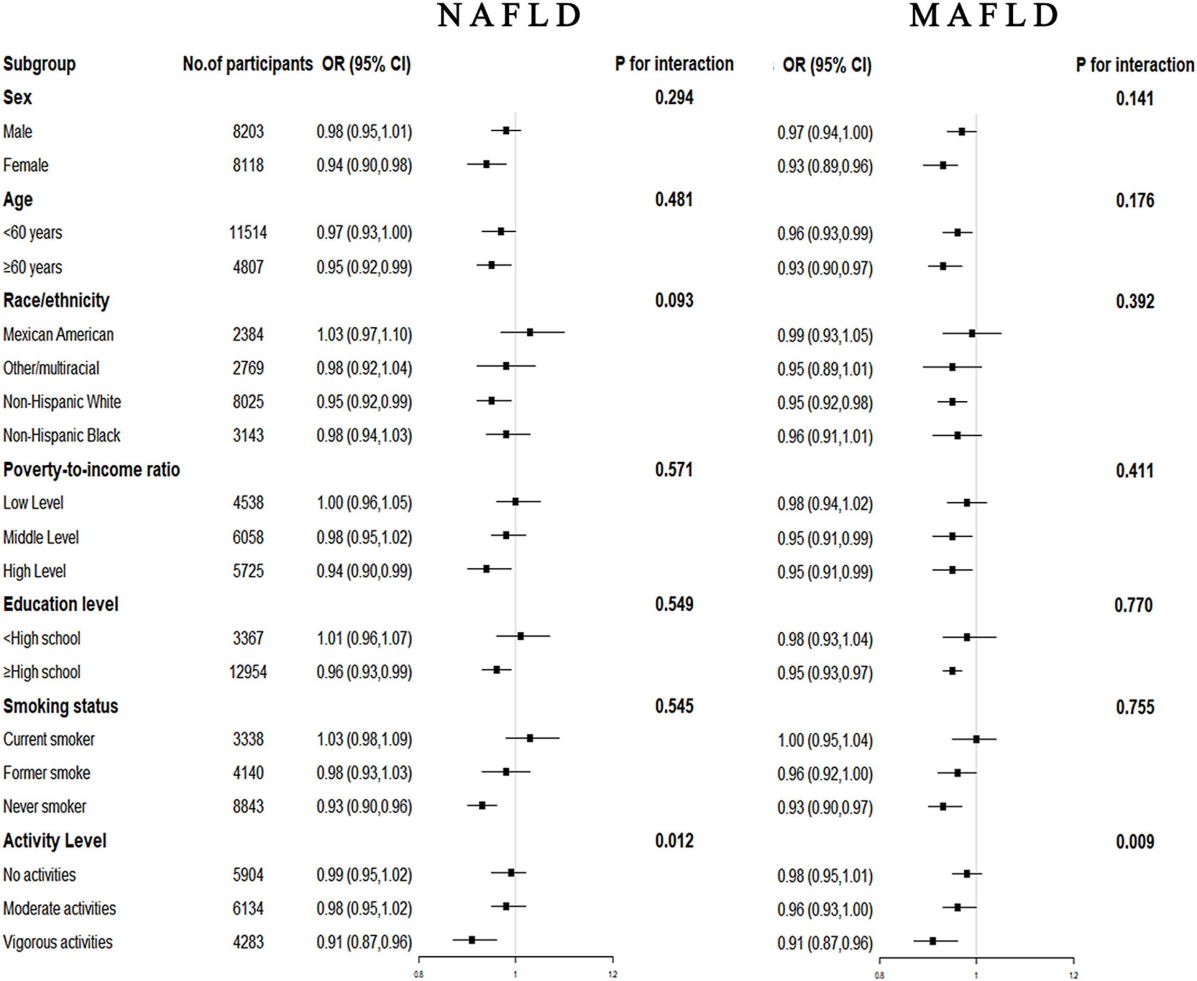
Forest plots for subgroup analysis. Subgroup analysis was stratified by age, sex, race, family income, education level, smoking status, and activity level. OR, odds ratio; CI, confidence interval; NAFLD, nonalcoholic fatty liver disease; MAFLD, metabolic-associated fatty liver disease.

Stratified analysis by age, sex, race, family income, education level, smoking status, and activity level is shown in Figure 4. The results showed a stable negative association between CDAI and the prevalence of NAFLD/MAFLD in most populations (*p* for interaction >0.05). However, we found a significant interaction between CDAI and activity level with the risk of NAFLD/MAFLD (*p* for interaction <0.05), suggesting that these associations were more pronounced among vigorous activity participants.

The total participants were further divided into two groups in each sex and performed weighted multivariable-adjusted logistic regression. As shown in Table 3, in females, CDAI was associated with the risk of NAFLD (OR, 0.94; 95% CI, 0.90–0.98, *p* = 0.005) and MAFLD (OR, 0.93; 95% CI, 0.98–0.96, *p* < 0.001) after adjusting for age, race, activity level, educational level, family income, energy intake, and smoking. However, the similar relationship was not significant in males.

**Table 3:** The adjusted odds ratio (OR) of NAFLD/MAFLD according to gender.

		Male OR (95% CI)^2^	*p* Value	Female OR (95% CI)	*p* Value
NAFLD	CDAI (continuous)	0.98 (0.95, 1.01)	0.191	0.94 (0.90, 0.98)	**0.005**
	Quartile of CDAI				
	Q1	Ref.^1^		Ref.	
	Q2	1.00 (0.96, 1.04)	0.616	1.00 (0.96, 1.05)	0.887
	Q3	0.97 (0.93, 1.02)	0.398	1.00 (0.95, 1.05)	0.943
	Q4	0.93 (0.89, 0.97)	0.575	0.95 (0.91, 1.00)	**0.028**
	*p* for trend	0.509		**0.036**	
MAFLD	CDAI (continuous)	0.97 (0.94, 1.00)	0.058	0.93 (0.98, 0.96)	**<0.001**
	Quartile of CDAI				
	Q1	Ref.		Ref.	
	Q2	1.00 (0.95, 1.05)	0.863	1.01 (0.95, 1.06)	0.833
	Q3	0.96 (0.91, 1.01)	0.112	1.00 (0.94, 1.06)	0.953
	Q4	0.96 (0.90, 1.03)	0.575	0.93 (0.88, 0.98)	**0.006**
	*p* for trend	0.113		**0.008**	

1 Ref: reference.

2 Model was adjusted for age, race, activity level, educational level, family income, energy intake, and smoking.

NAFLD, nonalcoholic fatty liver disease; MAFLD, metabolic-associated fatty liver disease; CDAI, composite dietary antioxidant index; OR, odds ratio; CI, confidence interval. The bold values signify statistical significance at *p* < 0.05.

### 3.6 Joint association and PAF of CDAI and PA status with NAFLD/MAFLD

**Figure 5:**
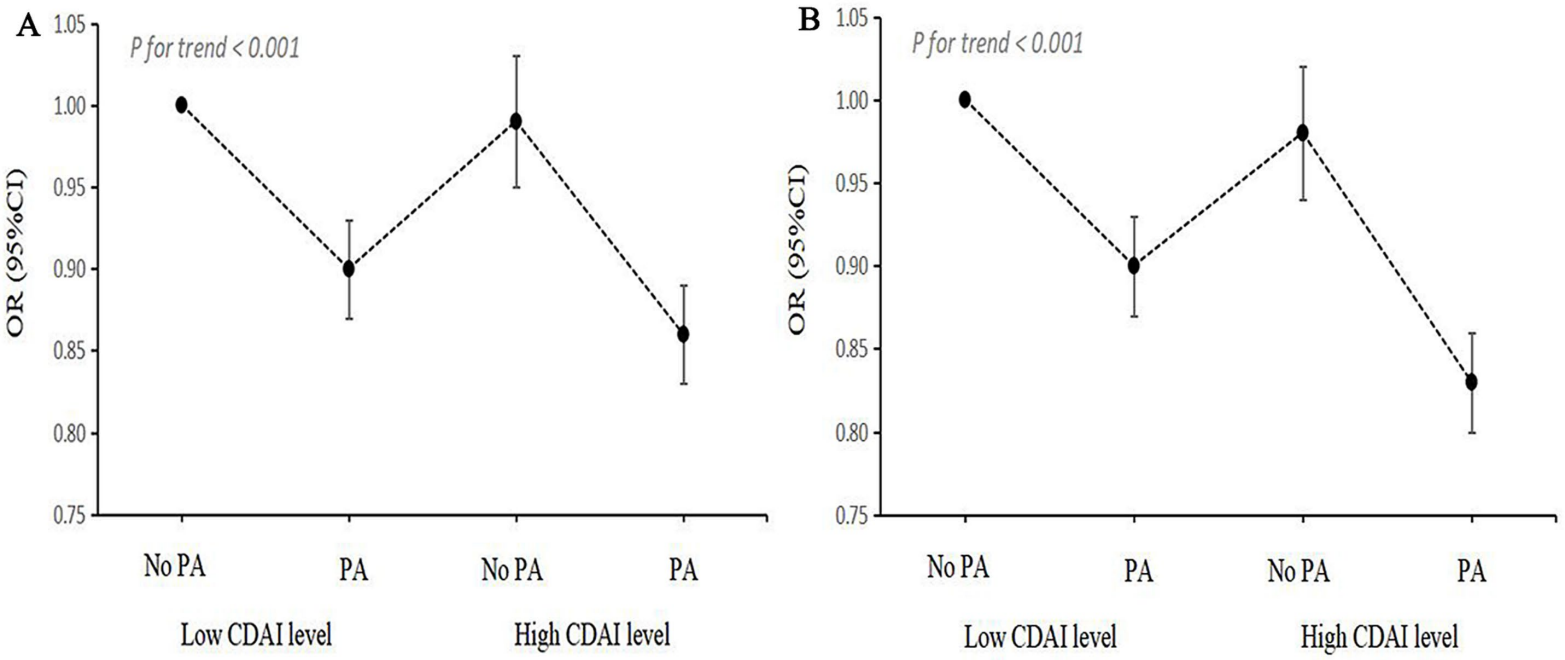
Joint association of CDAI levels and PA status with NAFLD/MAFLD. (A) NAFLD; (B) MAFLD. OR, odds ratio; CI, confidence interval; NAFLD, nonalcoholic fatty liver disease; MAFLD, metabolic-associated fatty liver disease; CDAI, composite dietary antioxidant index; PA, physical activity.

Since the interaction between CDAI and activity level with the risk of NAFLD/MAFLD was discovered, joint analysis was carried out. As shown in Figure 5 and Supplementary Table 7, participants with low levels of CDAI and inadequate PA had the highest risk of NAFLD/MAFLD in the fully adjusted model. Compared to the combination of low levels of CDAI and inadequate PA, the OR for NAFLD and MAFLD in the groups of low levels of CDAI and adequate PA were both 0.90 (95% CI, 0.87–0.93). Meanwhile, when compared with other groups, participants with high CDAI and adequate PA had the lowest risks of NAFLD (OR, 0.86; 95% CI, 0.83–0.89) and MAFLD (OR, 0.83; 95% CI, 0.80–0.86).

PAF analysis was performed to estimate the proportion of NAFLD/MAFLD risk among participants that could be mitigated by eliminating low CDAI and/or PA deficiency. As shown in Table 4, 5.2% of the reduction in NAFLD was attributed to high CDAI levels, while 25.0% was linked to adequate PA. Additionally, 26.4% of the reduction in NAFLD was associated with both high CDAI levels and adequate PA. For MAFLD, 4.8% of the reduction was attributed to high CDAI levels, 25.0% to adequate PA, and 26.9% to both high CDAI levels and adequate PA.

**Table 4:** PAF of CDAI levels and PA status with NAFLD/MAFLD.

		Model 1^1^ PAF (95% CI)	*p* Value	Model 2^2^ PAF (95% CI)	*p* Value	Model 3^3^ PAF (95% CI)	*p* Value
NAFLD	CDAI	−0.054 (−0.076, −0.033)	**<0.001**	−0.063 (−0.085, −0.041)	**<0.001**	−0.052 (−0.077, −0.027)	**<0.001**
	PA	−0.379 (−0.414, −0.344)	**<0.001**	−0.280 (−0.314, −0.246)	**<0.001**	−0.250 (−0.286, −0.215)	**<0.001**
	CDAI and PA	−0.384 (−0.433, −0.336)	**<0.001**	−0.301 (−0.348, −0.254)	**<0.001**	−0.264 (−0.314, −0.215)	**<0.001**
MAFLD	CDAI	−0.038 (−0.057, −0.018)	**<0.001**	−0.058 (−0.077, −0.038)	**<0.001**	−0.048 (−0.071, −0.025)	**<0.001**
	PA	−0.346 (−0.377, −0.314)	**<0.001**	−0.266 (−0.297, −0.236)	**<0.001**	−0.250 (−0.282, −0.218)	**<0.001**
	CDAI and PA	−0.327 (−0.370, −0.285)	**<0.001**	−0.273 (−0.314, −0.231)	**<0.001**	−0.269 (−0.313, −0.224)	**<0.001**

1 Model 1 was non-adjusted model.

2 Model 2 was adjusted for age, gender, and race.

3 Model 3 was further adjusted for educational level, family income, energy intake, and smoking.

PAF, population attributable fraction; CDAI, composite dietary antioxidant index; PA, physical activity; NAFLD, nonalcoholic fatty liver disease; MAFLD, metabolic-associated fatty liver disease; CI, confidence interval. The bold values signify statistical significance at *p* < 0.05.

## 4 Discussion

In this study, we utilized the nationally representative NHANES 2003–2018 dataset to investigate the relationship between CDAI and NAFLD/MAFLD. Our findings indicate that high CDAI levels are significantly associated with NAFLD/MAFLD prevalence, as evidenced by a 0.97-fold and a 0.95-fold decrease in the odds of NAFLD/MAFLD, respectively. Additionally, some inflammatory biomarkers, such as WBC, neutrophils, lymphocytes, and C-reactive protein, mediate this relationship. The relationship between CDAI and NAFLD/MAFLD is complex, influenced by numerous factors including age, sex, race, family income, PA, educational level, and smoking. Our findings reveal that combining effective PA with high CDAI levels is more effective in reducing the prevalence of NAFLD/MAFLD than high CDAI levels alone. These findings underscore the complex effects of dietary antioxidants on NAFLD/MAFLD and could guide future research that may inform subsequent clinical guidelines.

Dietary constituents play pivotal roles in NAFLD/MAFLD development. Prior studies on the effect of dietary antioxidants on NAFLD/MAFLD have been conducted, but significant controversy remains. Several studies have shown that vitamin A has a significant protective effect on the development of NAFLD (46, 47). A similar inverse association was also observed among people who had higher dietary vitamin C levels (48). The potential mechanism may be that higher vitamin C intake can restore gut-liver functions and antioxidant status (49). However, a retrospective study from Italy showed that the ingestion of vitamins A and C was greater in patients with nonalcoholic steatohepatitis than in controls (50). For vitamin E, controversies also persist across various studies. Some studies have suggested that vitamin E may improve the progression of NAFLD (47, 51). However, others contend that it has no significant impact on this disease (52). Minerals are also crucial in NAFLD/MAFLD development. A case–control study reported significantly lower zinc intake in NAFLD patients compared to controls (53), while this relationship was considered to be non-significant in a meta-analysis (54). Similarly, the impact of selenium on NAFLD remains inconclusive (55, 56). Some researches thought the selenoprotein produced by the liver may play a role in hepatic steatosis (57). Our analysis shows a negative association between vitamin E levels and NAFLD/MAFLD prevalence, while selenium levels are positively associated with the risk of NAFLD/MAFLD.

The dietary composition of the diet is multifaceted, and focusing solely on a single vitamin or mineral intake may not yield substantial benefits. CDAI is a comprehensive assessment of the overall impact of dietary antioxidants, but related research remains limited. Previous studies have investigated the effects of CDAI on the prevalence of MASLD with three-year sample sizes, whereas our study involved 16-year participants (29, 30). Both studies reported a negative relationship. However, Zhang et al. found that a high intake of CDAI was beneficial for decreasing prevalence in MASLD in a linear manner, while Yang et al. found a non-linear relationship. One reason for this difference may be the important role of PA in this relationship, PA was included as a covariate by Zhang et al., while it was not considered in another study. It is noteworthy that regular active PA has been shown to have beneficial effects on NAFLD development (58). Notably, A “triple-hit behavioral phenotype” has been introduced recently, highlighting the joint effects of sedentary behavior, low PA, and poor diet on NAFLD (45). Our findings on the joint association of CDAI and PA consolidate this phenotype, that compared to high CDAI levels alone, combining effective PA is significantly associated with more reduction in NAFLD/MAFLD prevalence. Our findings carry out a new conception to the research on the association between diet and NAFLD/MALD, emphasizing the necessity for further exploration into the joint association of diet and PA.

The etiology of NAFLD involves both genetic and environmental factors, and early studies suggested that the “double-hit” hypothesis can explain the development of this disease. Insulin resistance-induced hepatic steatosis occurs as the “first hit,” followed by oxidative stress-induced hepatocellular injury as the “second hit” (59). The “multi-hit” hypothesis, currently considered a more accurate explanation for the pathogenesis of NAFLD/MAFLD, encompasses lipotoxicity, mitochondrial dysfunction, oxidative stress, endoplasmic reticulum stress, and inflammatory cytokines (60). Oxidative stress is defined as an imbalance between cellular antioxidants and pro-oxidants, including ROS and reactive nitrogen species, resulting in cellular damage and leading to cell death (61). A strong association existed between NAFLD/MAFLD and oxidative stress levels. Upon oxidative stress, excessive ROS are produced, subsequently damaging the respiratory chain and causing oxidative harm to the mitochondrial genome in hepatocytes, which also leads to the production of additional ROS, thus resulting in a vicious cycle (62). Some studies have demonstrated that numerous natural products, which possess antioxidant and anti-inflammatory properties, may potentially provide preventive effects against hepatic steatosis in NAFLD (63). The association noted with dietary antioxidants and NAFLD in our study is also consistent with prior studies, importantly, the present study revealed a mediate effect of inflammatory biomarkers such as WBC, neutrophils, lymphocytes, and C-reactive protein. These results reinforce the evidence suggesting that oxidative stress and inflammatory play as crucial role in the pathogenesis of NAFLD/MAFLD. However, while inflammatory markers were found to partially mediate the association between CDAI and the disease, the specific biological mechanisms still require further in-depth investigation.

In addition, the subgroup analysis indicated a significant different effect between CDAI and NAFLD/MAFLD, suggesting that the protective effect of higher levels of CDAI concentration on NAFLD/MAFLD only in females and not males. Similarly, Yang et al. found that the relationship between CDAI and MASLD particularly significant in females (29). This may be attributed to the fact that sex hormones are influencing factors of NAFLD (64). Estrogen significantly influences the mitochondrial respiratory chain by regulating mitochondrial gene transcription. Meanwhile, excessive accumulation of estrogen can lead to increased generation of reactive oxygen species, exacerbating mitochondrial DNA mutations and damaging mitochondrial proteins (65). These findings support our findings regarding gender grouping and highlight the importance of increasing dietary antioxidant intake.

There are several strengths to our study. First, we ensured a sufficiently large sample size based on the NHANES database, using a complex multi-stage probability sampling methodology, ensuring the accurate representation of the non-institutionalized population and enhancing the generalizability of our findings. Secondly, we elucidated the mediating role of inflammatory biomarkers. More importantly, we observed a combined effect of CDAI and PA with NAFLD/MAFLD, underscoring the significance of both diet and PA in NAFLD/MAFLD managing. However, there are several limitations. First, self-reported recall bias concerning the CDAI might be present in observational studies. Second, there may also be confounding factors such as comorbidities not included in the study, which may have important potential impacts on the conclusions. Finally, as the data are limited to the US population, the results may exhibit bias and cannot be extrapolated to other demographic factors. Given the inherent constraints of cross-sectional studies, further validation from evidence-based research such as randomized controlled trials and longitudinal studies is warranted. Meanwhile, as the different effect of CDAI according to gender, further researches are needed to investigate the potential importance of estrogen in this relationship.

## 5 Conclusion

In summary, the current findings demonstrate that CDAI is negatively associated with NAFLD/MAFLD, with inflammation identified as a mediating factor. In addition, participants with characteristics of adequate PA and high levels of CDAI were more strongly correlated with the reduced prevalence of NAFLD/MAFLD. Therefore, promoting a dietary pattern rich in antioxidants combined with adequate physical activity may be an appropriate strategy for healthcare professionals to alleviate the burden and prevalence of NAFLD/MAFLD. Our study underscores the significance of both CDAI and PA in NAFLD/MAFLD managing, thereby offering valuable insight into this field. Additional studies in clinical cohorts are needed to confirm these findings.

## Data Availability

The original contributions presented in the study are included in the article/Supplementary material, further inquiries can be directed to the corresponding author.
