# Supplementary material for: Synergistic impact of Composite Dietary Antioxidant Index and physical activity on fatty liver disease
Source: Front Nutr. 2024 Nov 5;11:1486700. doi: 10.3389/fnut.2024.1486700 (PMC11573580; doi:10.3389/fnut.2024.1486700)
Supplement: Supplementary file 1 [file Data_Sheet_1.docx]

**Supplementary Table 1**

Baseline demographic characteristics of excluded and included data

| **Characteristic** | **Excluded Data**, N = 4,327 ^1^ | **Included Data**, N = 16,327 ^1^ | **Standardized Difference** ^2^ |
| --- | --- | --- | --- |
| Age (%) |  |  |  |
| <65 years | 3,196 (80%) | 12,911 (84%) | 0.10 |
| ≥65 years | 1,131 (20%) | 3,416 (16%) | 0.10 |
| Sex (%) |  |  |  |
| Female | 2,345 (53%) | 8,121 (50%) | 0.06 |
| Male | 1,982 (47%) | 8,206 (50%) | 0.06 |
| Income (%) |  |  |  |
| Low Level | 881 (23%) | 4,543 (19%) | 0.09 |
| Middle Level | 1,150 (40%) | 6,058 (34%) | 0.12 |
| High Level | 812 (37%) | 5,726 (47%) | 0.20 |
| Education level (%) |  |  |  |
| <High school | 1,344 (20%) | 3,370 (13%) | 0.18 |
| ≥High school | 2,983 (80%) | 12,957 (87%) | 0.18 |
| BMI (kg/m^2^) |  |  |  |
| <25.0 | 1,263 (33%) | 4,944 (33%) | 0 |
| Overweight | 1,398 (31%) | 5,593 (34%) | 0.06 |
| Obesity | 1,666 (36%) | 5,790 (34%) | 0.04 |
| Activity level (%) |  |  |  |
| No activities | 1,097 (31%) | 5,906 (30%) | 0.02 |
| Moderate activities | 1,029 (43%) | 6,136 (40%) | 0.06 |
| Vigorous activities | 657 (26%) | 4,285 (30%) | 0.09 |
| Smoking status (%) |  |  |  |
| Current smoker | 917 (25%) | 3,338 (21%) | 0.09 |
| Former smoker | 1,047 (24%) | 4,140 (26%) | 0.05 |
| Never smoker | 2,360 (51%) | 8,843 (53%) | 0.04 |
| ^1^median (IQR) for continuous; n (%) for categorical;  ^2^The difference between the groups divided by the pooled standard deviation; a value lower than 10% is interpreted as a meaningless difference. | | | |

**Supplementary Table 2**

Characteristics of study population according to quartiles of Composite Dietary Antioxidant Index

| **Characteristic** | **Composite Dietary Antioxidant Index** | | | |
| --- | --- | --- | --- | --- |
|  | **Quartile 1**, N = 988 ^1^ | **Quartile 2**, N = 854 ^1^ | **Quartile 3**, N = 842 ^1^ | **Quartile 4**, N = 829 ^1^ |
| Age (years) | 49 (32, 63) | 52 (34, 63) | 48 (33, 59) | 47 (33, 59) |
| Sex (%) |  |  |  |  |
| Female | 615 (64%) | 490 (59%) | 399 (52%) | 285 (29%) |
| Male | 373 (36%) | 364 (41%) | 443 (48%) | 544 (71%) |
| Poverty income ratio | 2.16 (1.22, 4.07) | 2.81 (1.49, 4.79) | 2.99 (1.73, 5.00) | 3.68 (1.81, 5.00) |
| Education level (%) |  |  |  |  |
| <High school | 259 (18%) | 179 (11%) | 138 (9.5%) | 127 (9.1%) |
| ≥High school | 729 (82%) | 675 (89%) | 704 (90%) | 702 (91%) |
| Race/ethnicity (%) |  |  |  |  |
| Mexican American | 145 (9.1%) | 148 (8.9%) | 119 (8.2%) | 128 (8.9%) |
| Non-Hispanic Black | 254 (14%) | 169 (9.4%) | 156 (8.5%) | 152 (8.4%) |
| Non-Hispanic White | 340 (62%) | 330 (67%) | 326 (68%) | 304 (65%) |
| Other/multiracial | 249 (15%) | 207 (15%) | 241 (15%) | 245 (17%) |
| BMI (kg/m^2^) |  |  |  |  |
| <25.0 | 247 (26%) | 212 (27%) | 235 (27%) | 244 (30%) |
| Overweight | 299 (29%) | 290 (33%) | 280 (34%) | 279 (35%) |
| Obesity | 442 (45%) | 352 (40%) | 327 (39%) | 306 (35%) |
| Activity level (%) |  |  |  |  |
| No activities | 585 (56%) | 465 (48%) | 415 (42%) | 340 (34%) |
| Moderate activities | 239 (26%) | 236 (33%) | 214 (28%) | 194 (23%) |
| Vigorous activities | 164 (18%) | 153 (20%) | 213 (29%) | 295 (43%) |
| Smoking status (%) |  |  |  |  |
| Current smoker | 254 (29%) | 149 (17%) | 167 (16%) | 130 (14%) |
| Former smoker | 238 (23%) | 210 (27%) | 220 (29%) | 221 (29%) |
| Never smoker | 496 (48%) | 495 (56%) | 455 (56%) | 478 (57%) |
| Energy (kcal) | 1,359 (1,077, 1,655) | 1,847 (1,540, 2,149) | 2,212 (1,873, 2,647) | 2,726 (2,134, 3,286) |
| WBC (1000 cells/uL) | 6.90 (5.60, 8.40) | 6.70 (5.60, 8.00) | 6.44 (5.50, 7.80) | 6.20 (5.20, 7.50) |
| Hb (g/dL) | 14.00 (13.20, 15.10) | 14.30 (13.40, 15.30) | 14.30 (13.40, 15.30) | 14.90 (13.90, 15.60) |
| PLT (1000 cells/uL) | 243 (208, 284) | 232 (196, 271) | 228 (196, 267) | 220 (191, 253) |
| GGT (IU/L) | 19 (13, 30) | 19 (13, 28) | 20 (14, 30) | 20 (15, 31) |
| TB (umol/L) | 6.8 (5.1, 10.3) | 8.6 (6.8, 12.0) | 8.6 (6.8, 12.0) | 10.3 (6.8, 12.0) |
| ALP (IU/L) | 69 (58, 87) | 69 (56, 82) | 66 (54, 81) | 65 (55, 79) |
| Hs-CRP (mg/L) | 2.3 (0.9, 5.1) | 1.8 (0.7, 4.9) | 1.7 (0.8, 3.9) | 1.4 (0.6, 3.0) |
| NAFLD | 325 (31%) | 288 (31%) | 256 (29%) | 245 (27%) |
| MAFLD | 364 (35%) | 320 (35%) | 307 (37%) | 290 (32%) |
| ^1^Median (IQR); n (unweighted) (%);  NAFLD, nonalcoholic fatty liver disease; MAFLD, metabolic-associated fatty liver disease; BMI, body mass index; WBC, white blood cell; Hb, hemoglobin; PLT, platelet; GGT, gamma glutamyl transferase; TB, total bilirubin; ALP, alkaline phosphatase; Hs-CRP, high sensitive C-reactive protein. | | | | |

**Supplementary Table 3**

Association of six components of composite dietary antioxidant index and NAFLD

| **Components** | **Model 1**^1^ **OR (95% CI)** | **P Value** | **Model 2**^2^ **OR (95% CI)** | **P Value** | **Model 3**^3^ **OR (95% CI)** | **P Value** |
| --- | --- | --- | --- | --- | --- | --- |
| log (Vitamin A) | 1.48 (1.12, 1.96) | **0.006** | 1.39 (1.04, 1.84) | **0.025** | 1.38 (1.05, 1.82) | **0.021** |
| log (Vitamin C) | 0.58 (0.72, 1.00) | 0.054 | 0.80 (0.68, 0.95) | **0.011** | 0.85 (0.72, 1.02) | 0.076 |
| log (Vitamin E) | 0.48 (0.35, 0.65) | **<0.001** | 0.46 (0.33, 0.65) | **<0.001** | 0.55 (0.38, 0.79) | **0.001** |
| log (Carotenoid) | 0.80 (0.69, 0.92) | **0.002** | 0.74 (0.63, 0.86) | **<0.001** | 0.76 (0.65, 0.88) | **<0.001** |
| log (Selenium) | 1.90 (1.25, 2.88) | **0.003** | 2.33 (1.51, 3.58) | **<0.001** | 2.76 (1.73, 4.40) | **<0.001** |
| log (Zinc) | 0.81 (0.54, 1.22) | 0.317 | 0.75 (0.49, 1.14) | 0.175 | 0.99 (0.61, 1.60) | 0.964 |
| ^1^Model 1 was non-adjusted model.  ^2^Model 2 was adjusted for age, gender, and race.  ^3^Model 3 was adjusted for age, gender, race, activity level, educational level, family income, energy intake, and smoking.  NAFLD, nonalcoholic fatty liver disease; OR, odds ratio; CI, confidence interval. | | | | | | |

**Supplementary Table 4**

Association of six components of composite dietary antioxidant index and MAFLD

| **Components** | **Model 1**^1^ **OR (95% CI)** | **P Value** | **Model 2**^2^ **OR (95% CI)** | **P Value** | **Model 3**^3^ **OR (95% CI)** | **P Value** |
| --- | --- | --- | --- | --- | --- | --- |
| log (Vitamin A) | 1.24 (0.96, 1.61) | 0.102 | 1.17 (0.89, 1.54) | 0.253 | 1.14 (0.87, 1.49) | 0.336 |
| log (Vitamin C) | 0.80 (0.69, 0.94) | **0.005** | 0.76 (0.64, 0.89) | **<0.001** | 0.81 (0.69, 0.95) | **0.012** |
| log (Vitamin E) | 0.48 (0.36, 0.65) | **<0.001** | 0.46 (0.34, 0.63) | **<0.001** | 0.46 (0.33, 0.64) | **<0.001** |
| log (Carotenoid) | 0.89 (0.78, 1.02) | 0.100 | 0.84 (0.72, 0.97) | **0.016** | 0.89 (0.77, 1.03) | 0.112 |
| log (Selenium) | 2.50 (1.63, 3.83) | **<0.001** | 2.98 (1.92, 4.62) | **<0.001** | 2.72 (1.72, 4.31) | **<0.001** |
| log (Zinc) | 0.97 (0.63, 1.50) | 0.889 | 0.82 (0.53, 1.27) | 0.379 | 0.86 (0.54, 1.37) | 0.530 |
| ^1^Model 1 was non-adjusted model.  ^2^Model 2 was adjusted for age, gender, and race.  ^3^Model 3 was adjusted for age, gender, race, activity level, educational level, family income, energy intake, and smoking.  MAFLD, metabolic-associated fatty liver disease; OR, odds ratio; CI, confidence interval. | | | | | | |

**Supplementary Table 5**

Threshold effect analysis of CDAI on NAFLD/MAFLD by the two-piecewise linear regression

|  | **Inflection Point** | **Adjusted OR (95% CI)** | **P Value** |
| --- | --- | --- | --- |
| NAFLD | ≤ -3.943 | 0.88 (0.74, 1.05) | 0.146 |
|  | > -3.943 | 0.97 (0.94, 0.99) | **0.017** |
|  | Log-likelihood ratio | 0.081 | |
| MAFLD | ≤ 2.792 | 0.94 (0.91, 0.97) | **<0.001** |
|  | > 2.792 | 0.97 (0.93, 1.01) | 0.116 |
|  | Log-likelihood ratio | **0.038** | |
| Adjusted for age, gender, race, activity level, educational level, family income, energy intake, and smoking.  NAFLD, nonalcoholic fatty liver disease; MAFLD, metabolic-associated fatty liver disease; CDAI, composite dietary antioxidant index; OR, odds ratio; CI, confidence interval. | | | |

**Supplementary Table 6**

The relationship between CDAI and inflammatory biomarkers

|  | **Continuous** | | **Q1^2^** | **Q2** | | **Q3** | | **Q4** | | ***P*_trend_** |
| --- | --- | --- | --- | --- | --- | --- | --- | --- | --- | --- |
|  | **OR (95% CI)** | **P** | **OR (95% CI)** | **OR (95% CI)** | **P** | **OR (95% CI)** | **P** | **OR (95% CI)** | **P** |  |
| WBC^1^ | 0.96 (0.94, 0.98) | **0.001** | Ref ^3^(1.00) | 0.89 (0.74, 1.07) | 0.206 | 0.75 (0.61, 0.91) | **0.004** | 0.56 (0.44, 0.71) | **<0.001** | **<0.001** |
| Neutrophils | 0.97 (0.96, 0.99) | **<0.001** | Ref (1.00) | 0.95 (0.82, 1.09) | 0.441 | 0.80 (0.69, 0.92) | **0.003** | 0.70 (0.59, 0.83) | **<0.001** | **<0.001** |
| Lymphocytes | 0.99 (0.98, 1.00) | 0.063 | Ref (1.00) | 0.95 (0.89, 1.01) | 0.130 | 0.96 (0.89, 1.03) | 0.215 | 0.85 (0.77, 0.93) | **<0.001** | **<0.001** |
| C-reactive protein | 0.99 (0.98, 1.00) | **0.011** | Ref (1.00) | 1.05 (0.94, 1.17) | 0.365 | 0.99 (0.87, 1.12) | 0.854 | 0.89 (0.82, 0.96) | **0.005** | **0.021** |
| ^1^Model was adjusted for age, gender, race, activity level, educational level, family income, energy intake, and smoking.  ^2^Q, quartile of CDAI.  ^3^Ref, reference.  CDAI, composite dietary antioxidant index; WBC, white blood cell; OR, odds ratio; CI, confidence interval. | | | | | | | | | | |

**Supplementary Table 7**

|  | | | **Model 1**^2^ **OR (95% CI)** | **P Value** | **Model 2**^3^ **OR (95% CI)** | **P Value** | **Model 3**^4^ **OR (95% CI)** | **P Value** |
| --- | --- | --- | --- | --- | --- | --- | --- | --- |
| NAFLD | CDAI low level | No PA | Ref.^1^ |  | Ref. |  | Ref. |  |
|  |  | PA | 0.87 (0.84, 0.89) | **<0.001** | 0.89 (0.87, 0.92) | **<0.001** | 0.90 (0.87, 0.93) | **<0.001** |
|  | CDAI high level | No PA | 1.01 (0.97, 1.05) | 0.627 | 0.99 (0.95, 1.03) | 0.542 | 0.99 (0.95, 1.03) | 0.494 |
|  |  | PA | 0.84 (0.81, 0.87) | **<0.001** | 0.85 (0.82, 0.88) | **<0.001** | 0.86 (0.83, 0.89) | **<0.001** |
|  |  | P for trend | **<0.001** |  | **<0.001** |  | **<0.001** |  |
| MAFLD | CDAI low level | No PA | Ref. |  | Ref. |  | Ref. |  |
|  |  | PA | 0.86 (0.84, 0.89) | **<0.001** | 0.89 (0.86, 0.92) | **<0.001** | 0.90 (0.87, 0.93) | **<0.001** |
|  | CDAI high level | No PA | 1.04 (1.00, 1.08) | 0.062 | 1.01 (0.97, 1.05) | 0.737 | 0.98 (0.94, 1.02) | 0.418 |
|  |  | PA | 0.83 (0.80, 0.86) | **<0.001** | 0.84 (0.81, 0.87) | **<0.001** | 0.83 (0.80, 0.86) | **<0.001** |
|  |  | P for trend | **<0.001** |  | **<0.001** |  | **<0.001** |  |
| ^1^Ref: reference.  ^2^Model 1 was non-adjusted model.  ^3^Model 2 was adjusted for age, gender, and race.  ^4^Model 3 was adjusted for age, gender, race, activity level, educational level, family income, energy intake, and smoking.  NAFLD, nonalcoholic fatty liver disease; MAFLD, metabolic-associated fatty liver disease; CDAI, composite dietary antioxidant index; PA, physical activity; OR, odds ratio; CI, confidence interval. | | | | | | | | |

Joint association of CDAI levels and PA status with NAFLD/MAFLD


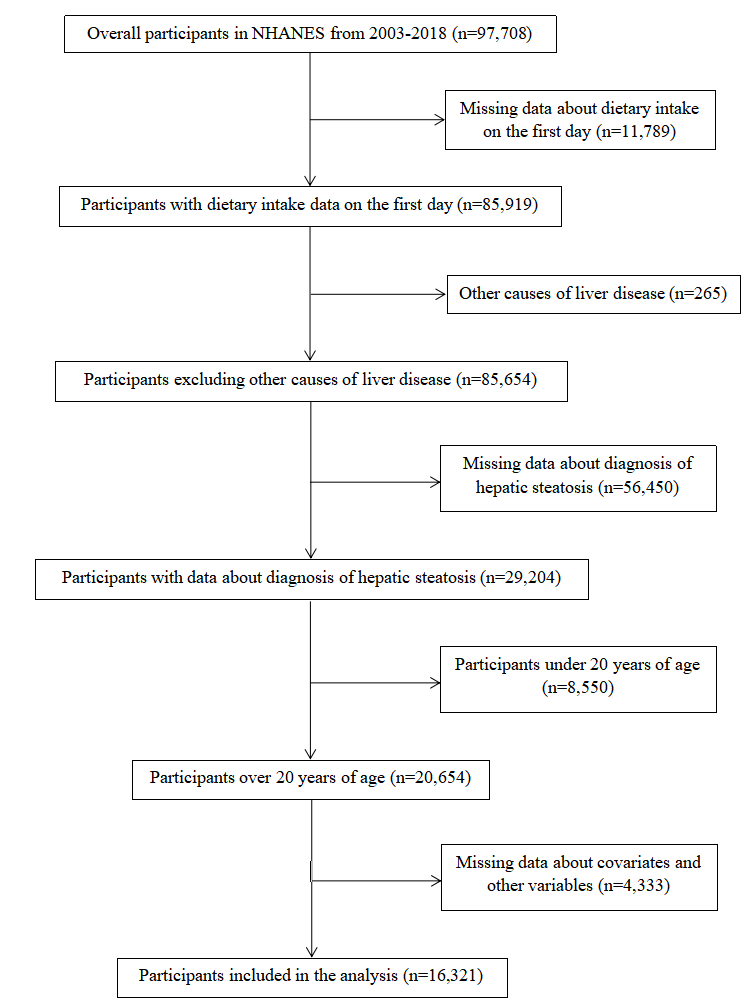


**Supplementary Figure 1.** Flowchart of sample selection in NHANES (2003-2018).

**
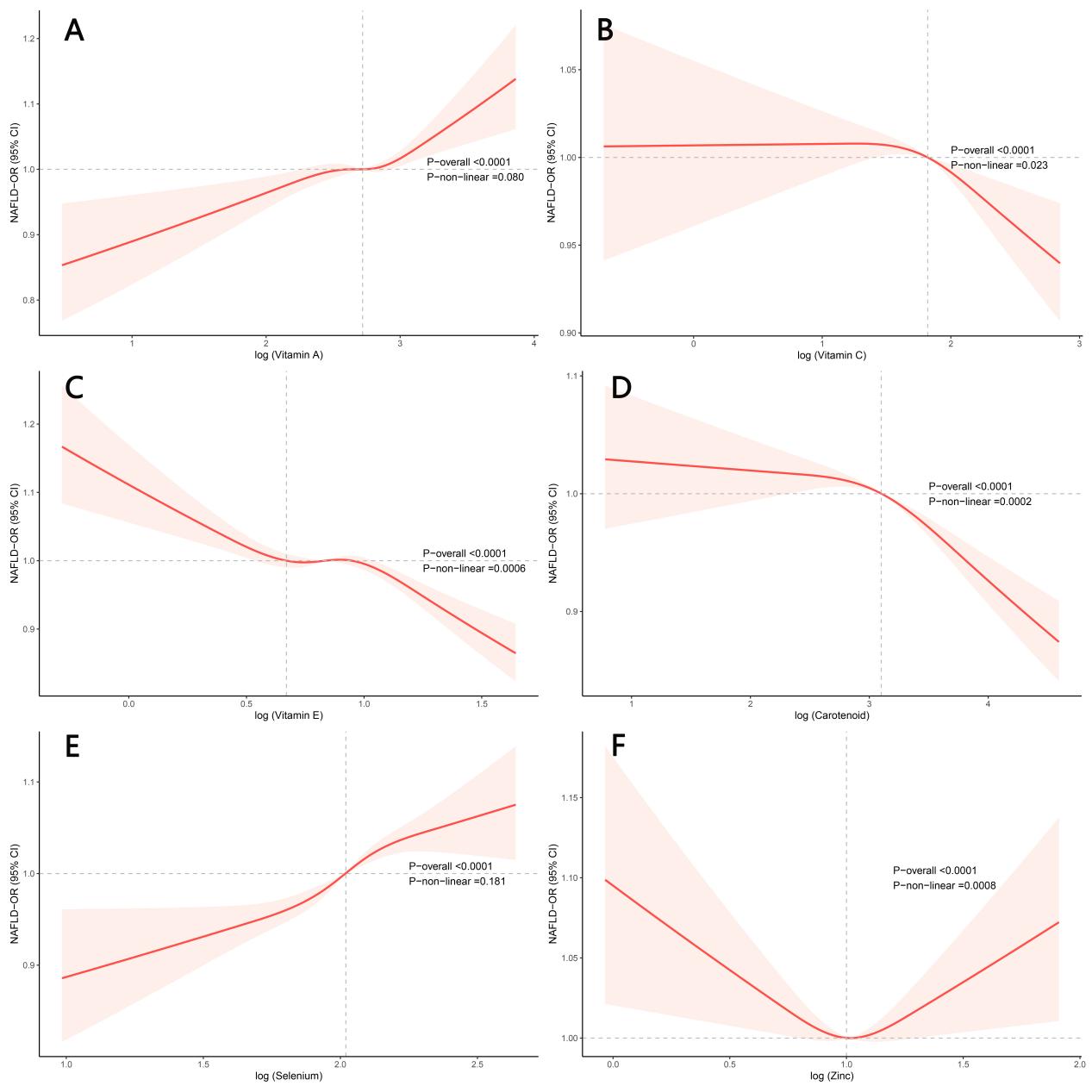
**

**Supplementary Figure 2.** Restricted cubic spline plots of the association between dietary antioxidant levels and NAFLD prevalence. (A) log(Vitamin A); (B) log(Vitamin C); (C) log(Vitamin E); (D) log(Carotenoid); (E) log(Selenium); (F) log(Zinc). Adjusted for age, gender, race, activity level, educational level, family income, energy intake, and smoking. OR, odds ratio; CI, confidence interval; NAFLD, nonalcoholic fatty liver disease.


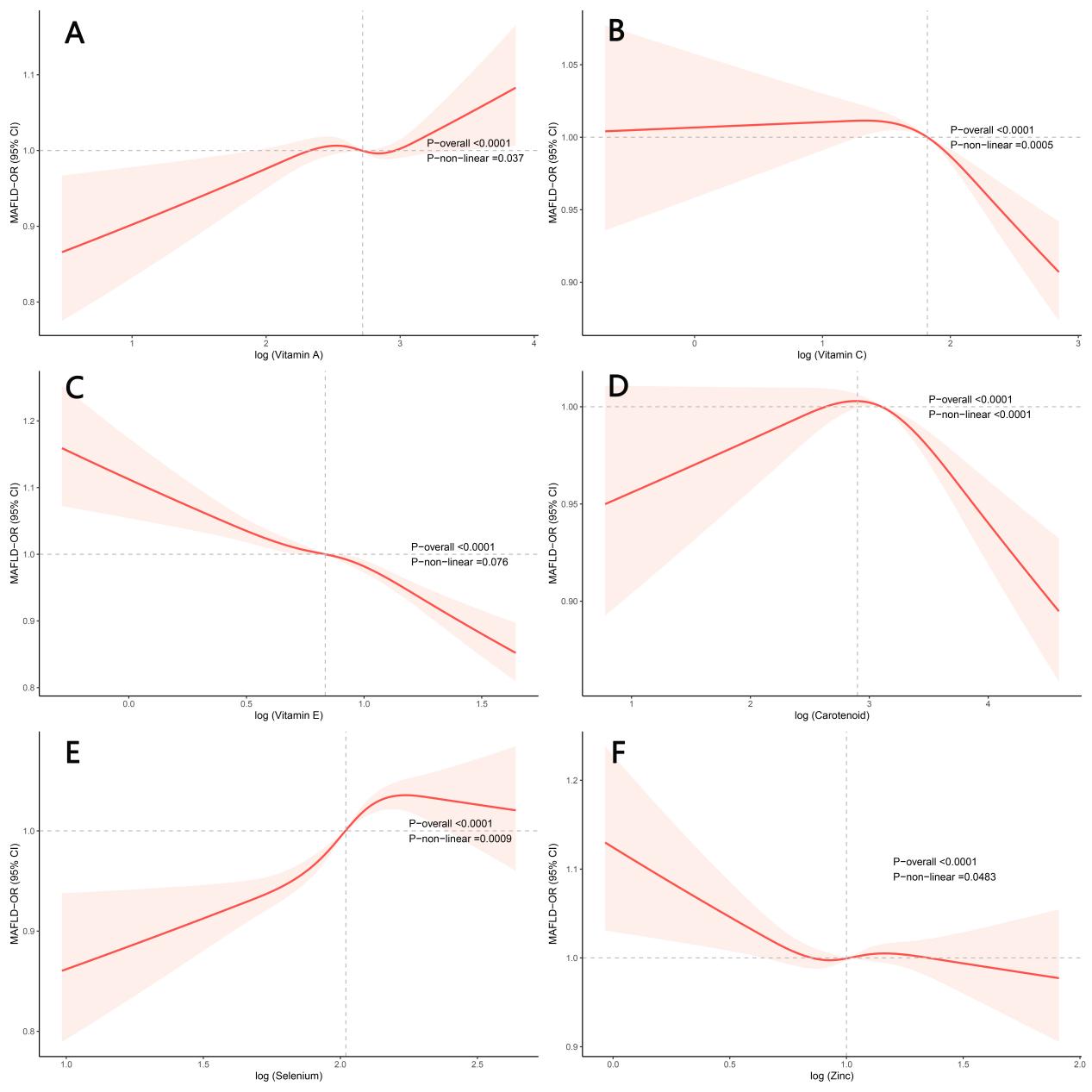


**Supplementary Figure 3.** Restricted cubic spline plots of the association between dietary antioxidant levels and MAFLD prevalence. (A) log(Vitamin A); (B) log(Vitamin C); (C) log(Vitamin E); (D) log(Carotenoid); (E) log(Selenium); (F) log(Zinc). Adjusted for age, gender, race, activity level, educational level, family income, energy intake, and smoking. OR, odds ratio; CI, confidence interval; MAFLD, metabolic-associated fatty liver disease.
